# Estimating geographic origins of corn and soybean biomass for biofuel production: A detailed dataset

**DOI:** 10.1016/j.dib.2024.110291

**Published:** 2024-03-05

**Authors:** Braden J. Limb, Jack P. Smith, Steven J. Simske, Jason C. Quinn

**Affiliations:** a Mechanical Engineering, Colorado State University, Fort Collins, CO 80523, USA; b B&D Engineering and Consulting LLC, 290 Amoretti St., Lander, WY 82520, USA; c Systems Engineering, Colorado State University, Fort Collins, CO 80523, USA

**Keywords:** Bioenergy, Biofuels, Corn ethanol, Soybean biodiesel, Geospatial biomass sourcing

## Abstract

Sustainable fuel initiatives in the United States such as the Environmental Protection Agency's Renewable Fuel Standard and the Department of Energy's Sustainable Aviation Fuel Grand Challenge have increased the production of corn ethanol and soybean biodiesel. However, the lack of precise information regarding biomass sourcing at a localized level has hindered accurate understanding of both biofuel costs and environmental impact of these production pathways. By harnessing the power of geospatial analysis and leveraging United States Department of Agriculture (USDA) crop census data, this dataset fills this critical knowledge gap. This dataset offers a novel estimation of geospatial biomass sourcing for biofuel production in the United States by synthesizing 2017 USDA crop census data, biorefinery data from the United States Energy Information Administration, and publicly available information about biomass sourcing for biofuel production. This dataset provides a detailed understanding of biomass use for first generation biofuel production, enabling stakeholders to make informed decisions about resource allocation, investment strategies, and infrastructure development. Furthermore, the county-level granularity of the dataset allows for increased fidelity in the techno-economic assessments and life-cycle analyses of first-generation biofuels in the United States.

Specifications Table

**Table:** 

Subject:	Bioenergy
Specific subject area:	Corn ethanol and soybean biodiesel feedstock sourcing.
Data format:	Analyzed
Type of data:	Table
Data collection:	This biomass sourcing data was estimated using custom-developed Python code and historical United States Department of Agriculture crop census data for corn and soybeans, biodiesel biorefinery data from the United States Energy Information Administration, and publicly available information about biomass sourcing for biofuel production. Biomass was aggregated to each biorefinery based on proximity to the plant (each biorefinery takes biomass from the nearest counties) and biorefinery priority was assigned based on biofuel production capacity, with larger facilities receiving biomass first.
Data source location:	Data Storage Institution: Colorado State University City/Town/Region: Fort Collins, Colorado Country: USA Input Data Sources Institution: United States Department of Agriculture, United States Energy Information Administration City/Town/Region: Washington, D.C. Country: USA
Data accessibility:	Repository name: Geospatial Dataset Estimating Corn and Soy Inputs for Biofuel Production Data identification number: 10.5281/zenodo.8250829 Direct URL to data: https://doi.org/10.5281/zenodo.8250829
Related research article:	Smith JP, Limb BJ, Beal CM, Banta KR, Field JL, Simske SJ, et al. Evaluating the sustainability of the 2017 US biofuel industry with an integrated techno-economic analysis and life cycle assessment. Journal of Cleaner Production 2023:137364. https://doi.org/10.1016/j.jclepro.2023.137364. [1]

## 1 Value of the Data

• This dataset provides a first-of-its-kind estimation of geospatial county-level biomass sourcing for biofuel production in the United States for the 2017 USDA crop census data.
• The dataset was created using the Python programing language, historical USDA crop census data, biodiesel biorefinery data from the US EIA, and publicly available information about biomass sourcing for biofuel production.
• Biofuels researchers and stakeholders can benefit from and reuse this dataset to evaluate domestic supply chain risks and more accurately calculate biofuel costs and environmental impact.
• This dataset provides an aggregated list of biodiesel facilities and the feedstock compositions which they use. This aggregated list is critical to maintaining an accurate supply chain map and identifying ideal locations for additional biorefineries based on biofuel demands and biofeedstock availabilities.

## 2 Data Description

The dataset described in this article provides a first-of-its-kind geospatial estimation for the corn and soybean biomass used for biofuel production (corn ethanol and soybean biodiesel, respectively) for the 2017 USDA crop census data. This dataset is publicly available on Zenodo and is titled *Geospatial Dataset Estimating Corn and Soy Inputs for Biofuel Production* [2]. The Python code used to generate this dataset is also provided publicly available on Zenodo and is titled *biofuel-feedstock-inputs: Code Publication* [3].

The dataset is broken up into two main .zip folders, one for corn ethanol and one for soybean biodiesel. Within each of these .zip folders a consistent structure is maintained. Three .csv data files and two .pdfs are provided. Due to the symmetry between the two datasets, they will be described together. When referring to filenames, {biofuel type} will be used in place of ethanol and biodiesel and {crop} will be used in place of corn and soybean.

The main datafile associated with this dataset are the ‘{biofuel type}_{crop}_use_by_county.csv’ files. These files provide the data for the county level biomass used for biofuel production. A description of the variables used in these files are presented in Table 1.

**Table 1:** Variable names, units and descriptions for data files titled ‘ethanol_corn_use_by_county.csv’ and ‘biodiesel_soybean_use_by_county.csv’.

Variable Name	Units	Description
FIPS	N/A	Federal Information Processing System (FIPS) Codes for identifying each county
total_kg	kilograms	Total corn or soybean grain produced in each county according to 2017 USDA census data
available_kg	kilograms	Assumed corn or soybean grain available in each county for biofuel production (90% of total)
used_kg	kilograms	Corn or soybean grain used for biofuel production in each county
remaining_kg	kilograms	Corn or soybean grain not used for biofuel production in each county
percent_used	Percent (%)	Percent of corn or soybean grain in each county used for biofuel
mean_distance_ crow_flys_mi	miles	The mean transportation distance for biomass used for biofuel production in the county as measured by the direct line distance
mean_distance_ freight_mi	miles	The mean transportation distance for biomass used for biofuel production in the county taking into non-direct distance for train and barge transport
transport_method	N/A	Transportation method used to transport biomass from the county to the biorefineries
plant_ids	N/A	Biorefinery identification number(s) for plants who use the county's biomass

The second datafile associated with this dataset are the ‘{biofuel type}_plant_information.csv’ files. These files provide the information about ethanol and biodiesel biorefineries including ID number, name, location, size, and feedstock transportation method. A description of the variables used in both files are presented in Table 2 and a description of biodiesel-specific variables are presented in the ‘biodiesel_plant_information.csv’ are presented in Table 3.

**Table 2:** Variable names, units and descriptions for data files titled ‘ethanol_plant_information.csv’ and ‘biodiesel_plant_information.csv’.

Variable Name	Units	Description
ID Number	N/A	Identification number of the biorefinery
Company	N/A	Parent company who owns the biorefinery
Site	N/A	Name of the biorefinery
State	N/A	The 2-letter (standard USPS) abbreviation for the state which the biorefinery resides
PADD	N/A	The Petroleum Administration for Defense Districts (PADD) designator as listed by the US Energy Information Administration
Cap_Mmgal	Millions of gallons of biofuel per year	Biorefinery's production capacity
Longitude	Degrees	Longitude of the location of the biorefinery as measured in degrees
Latitude	Degrees	Latitude of the location of the biorefinery as measured in degrees
Feedstock Transport	N/A	Transportation method used to transport biomass to the biorefinery

**Table 3:** Variable names, units and descriptions for variables included in the data files titled ‘biodiesel_plant_information.csv’ and excluded from ‘ethanol_plant_information.csv’.

Variable Name	Units	Description
Feedstock	N/A	Feedstock composition as defined by the sources listed in the ‘Reference’ column
Soybean Feedstock	N/A	Portion of the biorefinery feedstock which contains soybeans
Reference	N/A	Reference number where the feedstock information was acquired. Reference information is provided in the ‘References.pdf’ file

The third datafile associated with this dataset are the ‘{biofuel type}_validation.csv’ files. These files provide data on the accuracy of the estimated biomass grain used in each state for biofuel production to the publicly available reported values. A description of the variables used in these files are presented in Table 4.

**Table 4:** Variable names, units and descriptions for data files titled ‘ethanol_validation.csv’ and ‘biodiesel_validation.csv’.

Variable Name	Units	Description
State	N/A	Name of the state
Reported	Percent (%)	The reported percent of corn or soybean in the state that is used for corn ethanol or soybean biodiesel
Calculated	Percent (%)	The calculated percent of corn or soybean in the state that is used for corn ethanol or soybean biodiesel
Difference	Percent (%)	The difference between the reported and calculated percent of corn or soybean in the state that is used for corn ethanol or soybean biodiesel
National {crop} (%)	Percent (%)	The percent of the United States’ corn or soybean production that comes from the state
Reference	N/A	Reference number where the reported value was acquired. Reference information is provided in the ‘References.pdf’ file.

The two .pdf files provide supplementary information to the data. The ‘References.pdf’ file provides source information for the references identified in the ‘{biofuel type}_plant_information.csv’ and ‘{biofuel type}_validation.csv’ files. The ‘Data Dictionary – {crop} {biofuel type}.pdf’ files provide a data dictionary for the data files listed in this dataset. The data dictionary information is similar information to that listed in this section, but in an easier to access format when viewing the data.

## 3 Experimental Design, Materials and Methods

The Python programming language was used to generate this dataset using existing data from the United States’ Department of Agriculture (USDA) and United States Energy Information Administration (US EIA) [4], [5], [6]. The code is publicly available on Zenodo titled *biofuel-feedstock-inputs: Code Publication* [3]. Details on the calculations performed are presented in the following sections.

### 3.1 Estimation of biomass used for biofuel in each county

Since the primary research [1] focused on the county-level economic and environmental impacts of biofuel production across the US, it was important to know the amount of biomass produced in each county used for first generation biofuel production. However, no public information could be found on county-level crop use and only limited information was available on state-level crop use, especially for biofuel production.

As such, the county-level biomass used for biofuel production was estimated based on the geographic locations of biofuel plants. The US EIA database of ethanol and biodiesel plants was used for plant locations [5,6]. The USDA's National Agricultural Statistics Service was used for county-level biomass production from the 2017 USDA crop census [4]. The 2017 USDA crop census data was used for this analysis as it is the most recent USDA crop census data available and annual USDA survey data often has data discrepancies in geographic areas important for this research. County-level biomass was allocated to each biofuel plant based its proximity to the plant. For transportation purposes, it was assumed that all biomass within a county was located at the county's centroid. GIS data for each county was provided by Esri's USA Counties shapefile data and the centroid of each county was estimated using QGIS’ vector geometry tools [7,8]. The distance between each county's centroid and each biofuel plant was calculated using geodesic distance within the GeoPy Python module [9].

Each plant was assumed to take available biomass from the nearest counties. Plant priority was assigned based on biofuel production capacity, with larger facilities receiving biomass first. Since no information could be found on county-level biomass allocations, it was assumed that a maximum of 90% of county-wide biomass was available for biofuel production. A sensitivity analysis was performed to assess this assumption. Results from the sensitivity show this assumption had minimal impact on end results. For example, if the maximum biomass available in each county was reduced to 75%, the mean national biomass transportation distance only increased from 40.7 miles to 43.3 miles.

After biomass was allocated to every plant, the weighted mean transportation distance was calculated for each county and biofuel plant. The biomass quantity and transportation distance for each county was used to calculate the respective county's minimum fuel selling price and greenhouse gas intensity impacts in the primary study [1]. Specific methods related to ethanol and biodiesel plants are presented in the following sections.

### 3.2 Ethanol plants

The ethanol plants database from EIA includes 192 plants in the US with a maximum annual production capacity of 17.4 billion gallons. The national ethanol production in 2017 was 15.9 billion gallons [10]. Therefore, for county-level biomass estimation each ethanol plant was assumed to operate at a uniform 91.7% capacity (15.9/17.4). The conversion factor of 0.11 gal EtOH/kg corn gain was used to calculate the required biomass per plant [1]. Therefore, 143.5 million tonnes of corn grain were required to produce 15.9 billion gallons of ethanol in 2017. All plants with adequate corn grain in the surrounding counties were assumed to use trucks to transport the corn to each facility.

Five of the ethanol plants are in regions with limited corn grain supply. Four plants were in California (Aemetis Advanced Fuels Keyes Inc, Calgren Renewable Fuels LLC, Parallel Products, and Pelican Renewables LLC) and one is in Oregon (Alto Columbia LLC). To fulfill this demand, it was assumed that the required corn was transported via train from the Midwest [11]. The Midwest locations which supply corn to these plants were manually selected based on corn availability, rail accessibility, and rail routes that travel to the destination plants.

All corn for California was assumed to be provided via a rail network node at 40.023° latitude and -91.155° longitude in Adams County, Illinois. Counties surrounding this node provided enough corn grain to fulfill California's demand. Railway distances from this location were manually estimated to be 1900 miles, 1800 miles, 1700 miles, and 1860 miles for the Aemetis Advanced Fuels Keyes Inc, Calgren Renewable Fuels LLC, Parallel Products, and Pelican Renewables LLC ethanol plants; respectively.

Corn for the Oregon ethanol plant was assumed to be provided via a rail network node at 46.893° latitude and -99.066° longitude in Stutsman County, North Dakota. Stutsman County was able to fulfil the entire Oregon ethanol plant's corn demand. The railway distance from this location was manually estimated to be 1200 miles to the Oregon plant.

**Fig. 1:**
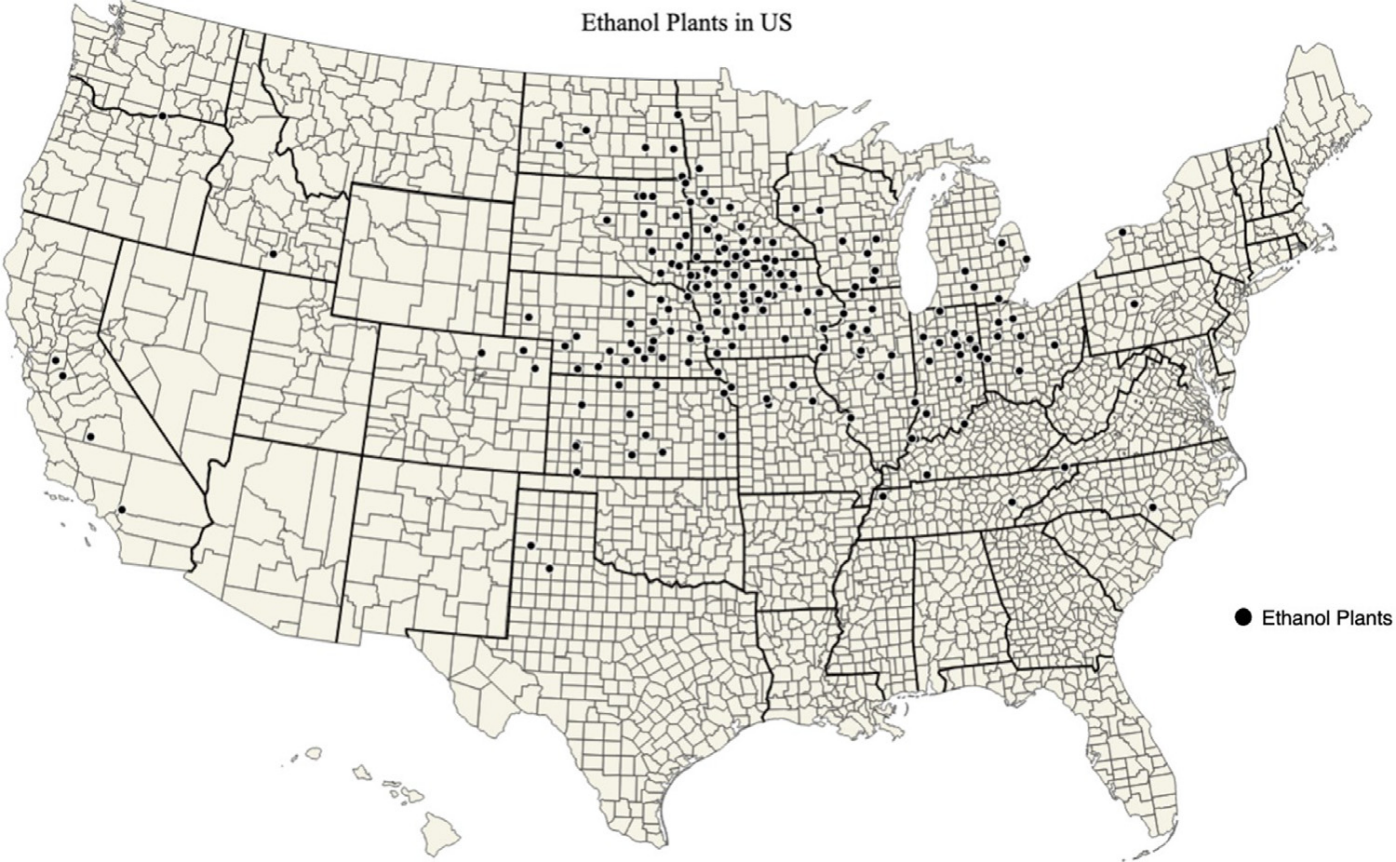
Map of US ethanol plants.

**Fig. 2:**
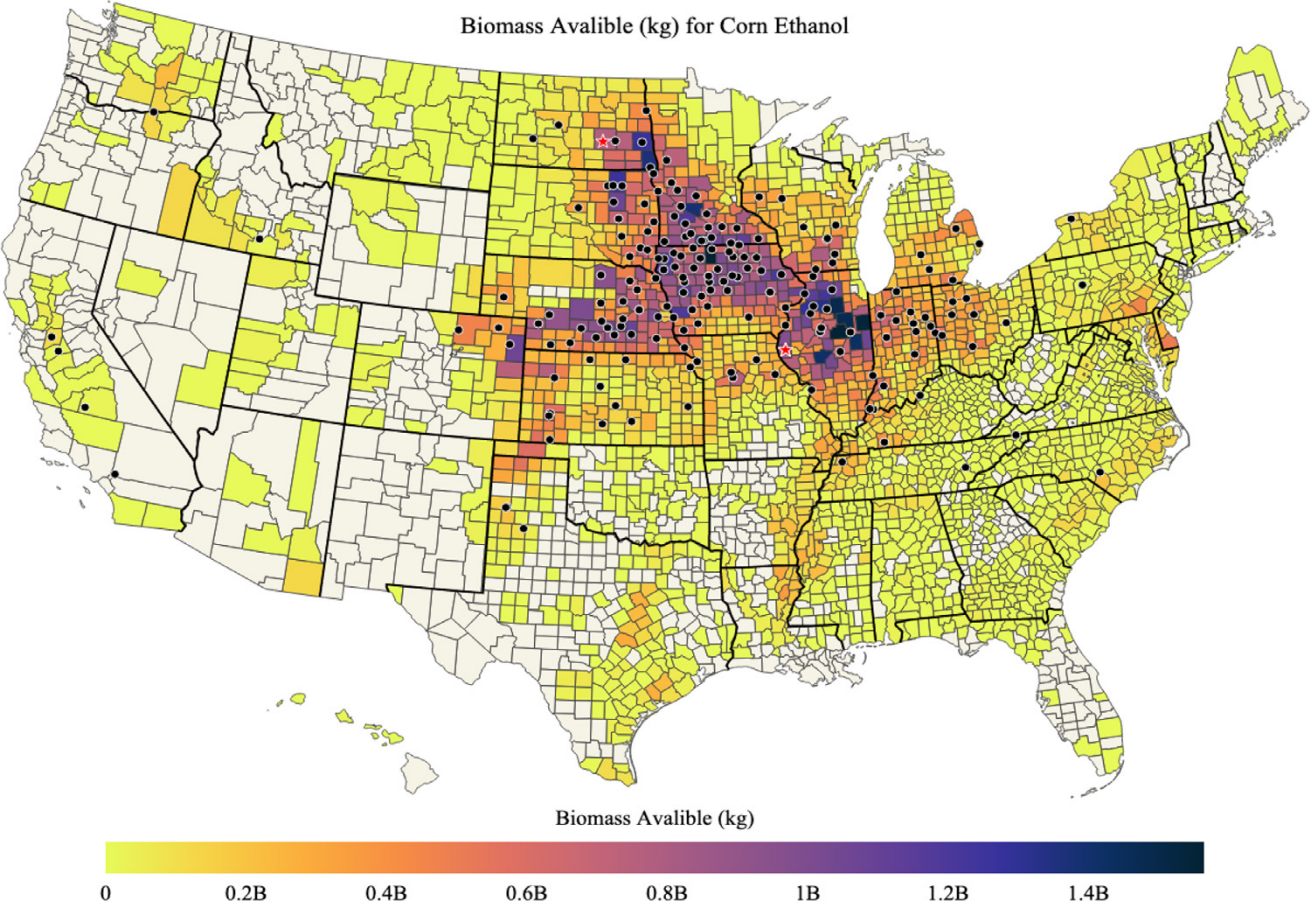
Available corn biomass (kg) in each county of the US.

**Fig. 3:**
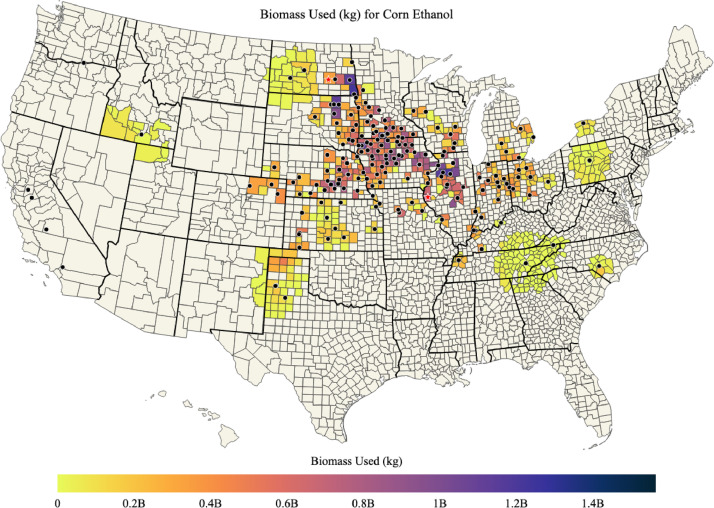
Total corn biomass (kg) used for corn ethanol in each county of the US.

**Fig. 4:**
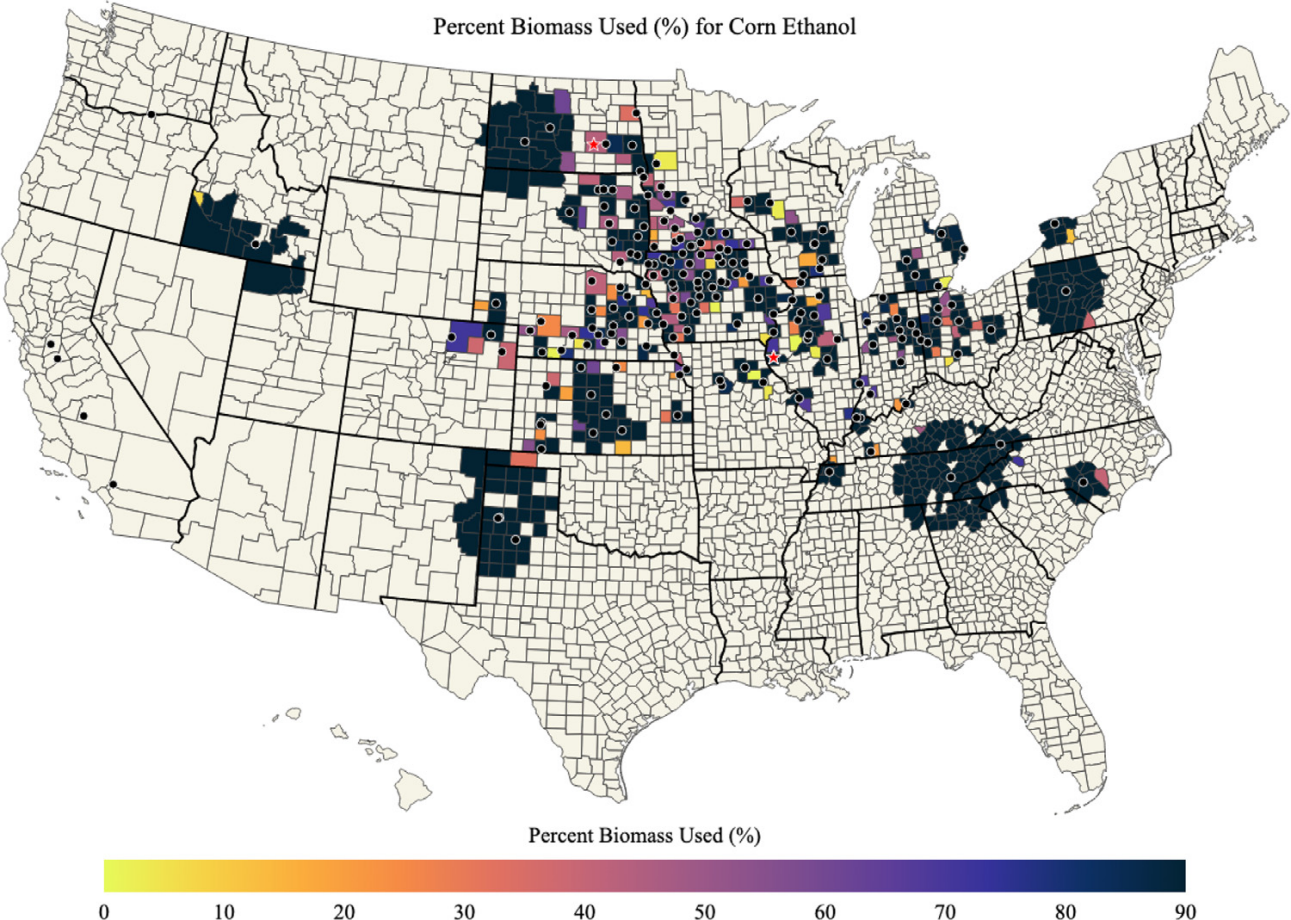
Percent of corn biomass used for corn ethanol in each county of the US.

**Fig. 5:**
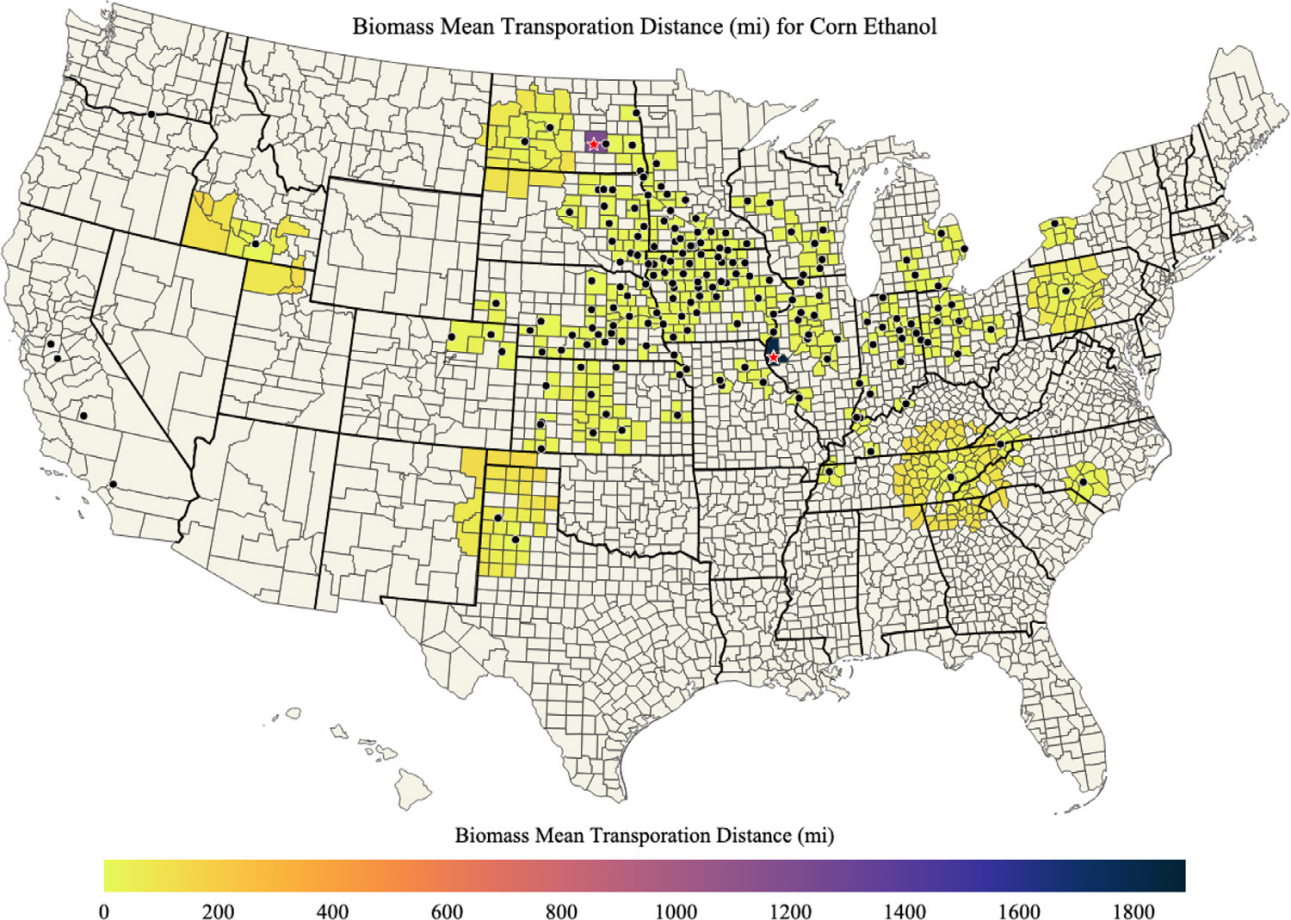
Mean transportation distance (mi) for corn used for corn ethanol in each county.

A list of ethanol plants is provided in ‘ethanol_plant_information.csv’ data file and map of ethanol plants is shown in Fig. 1. Maps of corn biomass available, corn biomass used for corn ethanol, percent of corn biomass used for corn ethanol, and the mean transportation distance for corn biomass used for corn ethanol in each county of the US are shown in Fig. 2, Fig. 3, Fig. 4, Fig. 5; respectively. Red stars on each of the maps designates the origin locations chosen for train transport of corn biomass to the California and Oregon ethanol plants.

### 3.3 Biodiesel plants

The biodiesel plants database from EIA includes 69 plants in the US with a maximum production capacity of 2.3 billion gallons. However, each biodiesel plant has a unique feedstock which could include soy oil, corn oil, vegetable oil, waste cooking oil, animal fats, etc. Therefore, only a portion of the biodiesel plants produce soybean-based biodiesel. The national soybean biodiesel production in 2017 was 878 million gallons [12]. Unfortunately, there isn't a single database of feedstock information for all biodiesel plants. Biodiesel Magazine hosts a database of current biodiesel plants, but most entries lack updated information on feedstock composition [13]. However, previous versions of the Biodiesel Magazine plant database had feedstock compositions [14]. As such, the Wayback Machine was used to access historical versions of the Biodiesel Magazine database to gather feedstock information for each plant [15]. All historical records were pulled between September and December 2017. For biodiesel plants that were not in the Biodiesel Magazine database, lacked feedstock information, or were listed as “Multi-Feedstock” plants; additional research was done to identify if soybeans were part of the feedstock composition. Company websites or local publications typically provided enough information to verify if soybeans were or were not used as a biodiesel feedstock. A full list of biodiesel plants, feedstock compositions, and references is provided in the ‘biodiesel_plant_information.csv’ data file.

**Fig. 6:**
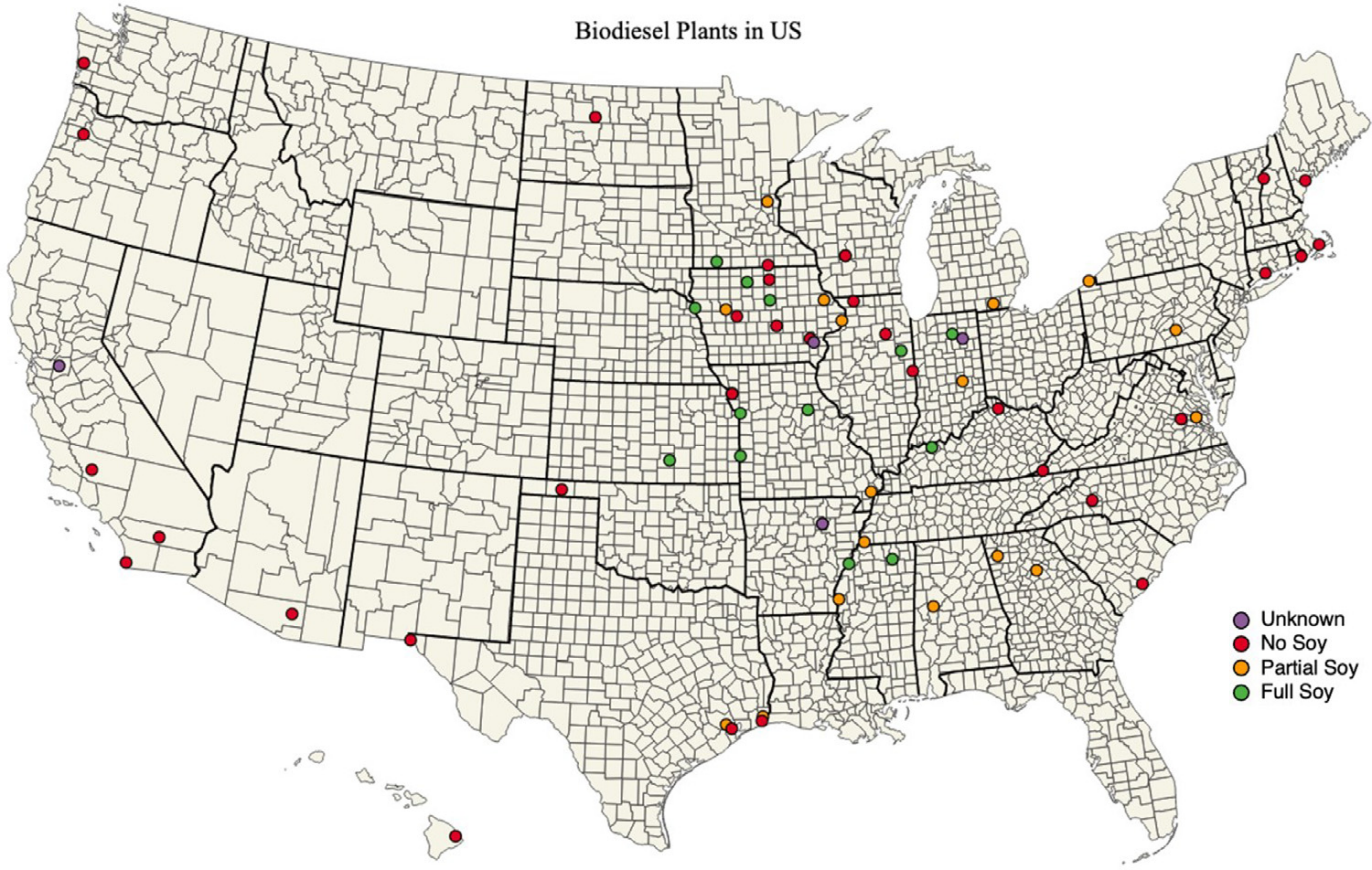
Map of US biodiesel plants specifying if feedstock includes soybeans.

In total, 14 plants (799 million gallons) used soybeans as the only feedstock, 18 plants (577 million gallons) used soybeans as a partial feedstock, 33 plants (832 million gallons) did not use soybeans as feedstock, and the feedstock of 4 plants (48 million gallons) could not be identified and were assumed to not use soybeans as feedstock. A map of US biodiesel plants is shown in Fig. 6.

It was assumed that the soy-only biodiesel plants would operate at 95% capacity factor to account for downtime and maintenance [16]. This results in 759 million gallons of soybean biodiesel production. Therefore, this equals a shortfall of 119 million gallons of soybean biodiesel to meet 2017 production levels. As such, the biodiesel plants identified as having a partial soybean feedstock were assumed to meet this 119 million gallon shortfall. This assumption requires that these 18 partial soy plants need to produce 20.6% of their maximum biodiesel capacity from soybeans. The conversion factor of 0.06 gal biodiesel/kg soybean was used to calculate the required biomass per plant [1]. In total, 14.4 million tonnes of soybeans were required to produce 878 million gallons of soybean biodiesel in 2017.

Two of the biodiesel plants were located in Texas where limited soybeans was available for biodiesel production. As such, it was assumed that soybeans for these plants were transported via barge down the Mississippi River [17]. The locations selected to supply soybeans to these plants was manually selected based on soybean availability and barge port accessibility. All soybeans were assumed to be provided via Port of Rosedale, Mississippi at 33.813° latitude and -91.022° longitude in Bolivar County, Mississippi. Counties surrounding this port provided enough soybeans to fulfill the demand of both Texas plants. Barge distances from the port were manually estimated to be 800 miles and 830 miles to RBF Port Neches LLC and World Energy Biox Biofuels LLC biodiesel plants, respectively.

**Fig. 7:**
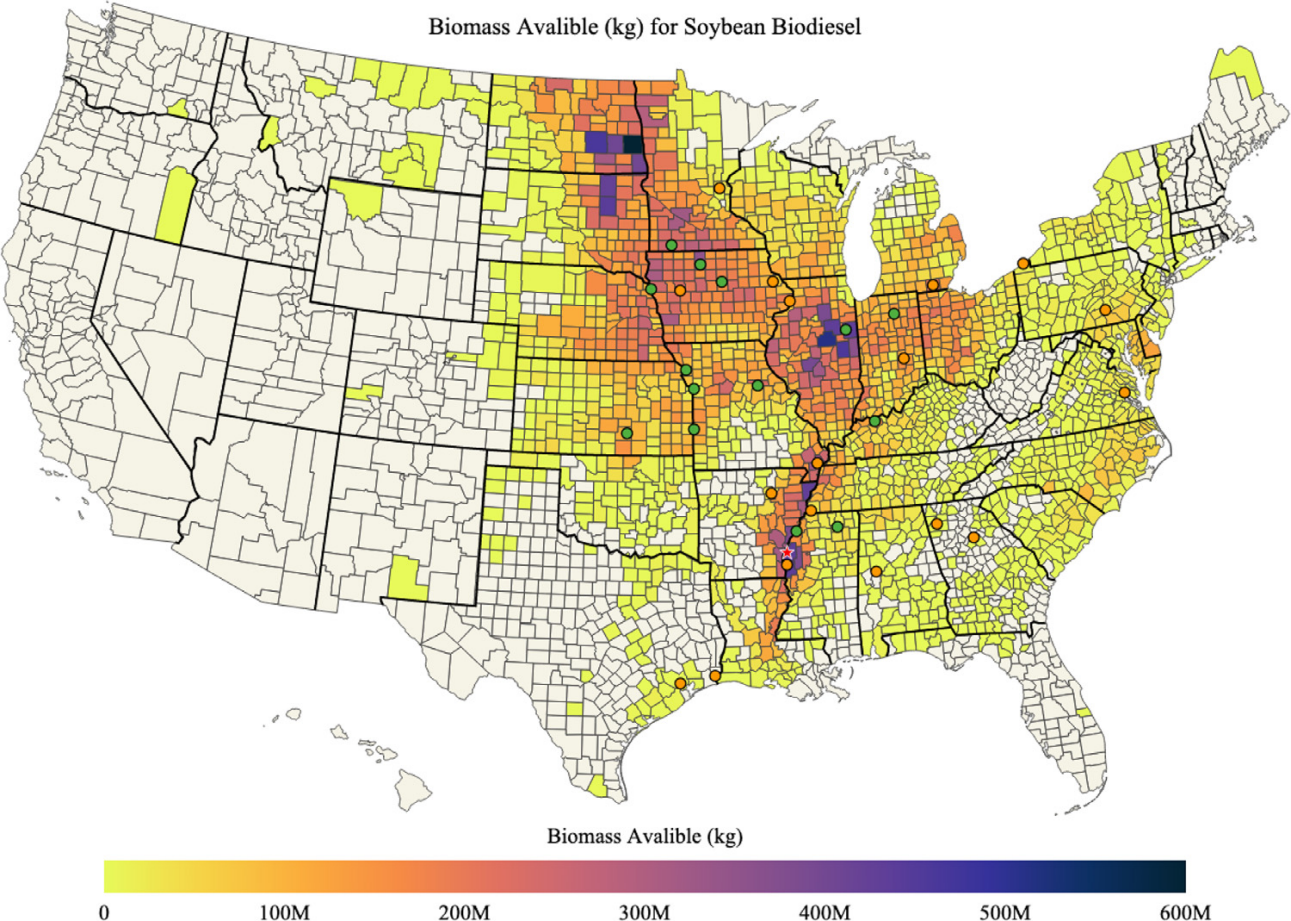
Available soybean biomass (kg) in each county of the US.

**Fig. 8:**
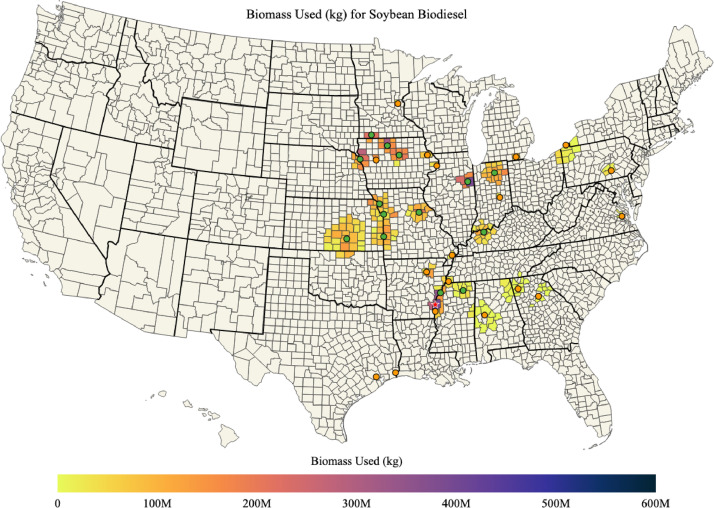
Total soybean biomass (kg) used for soybean biodiesel in each county of the US.

**Fig. 9:**
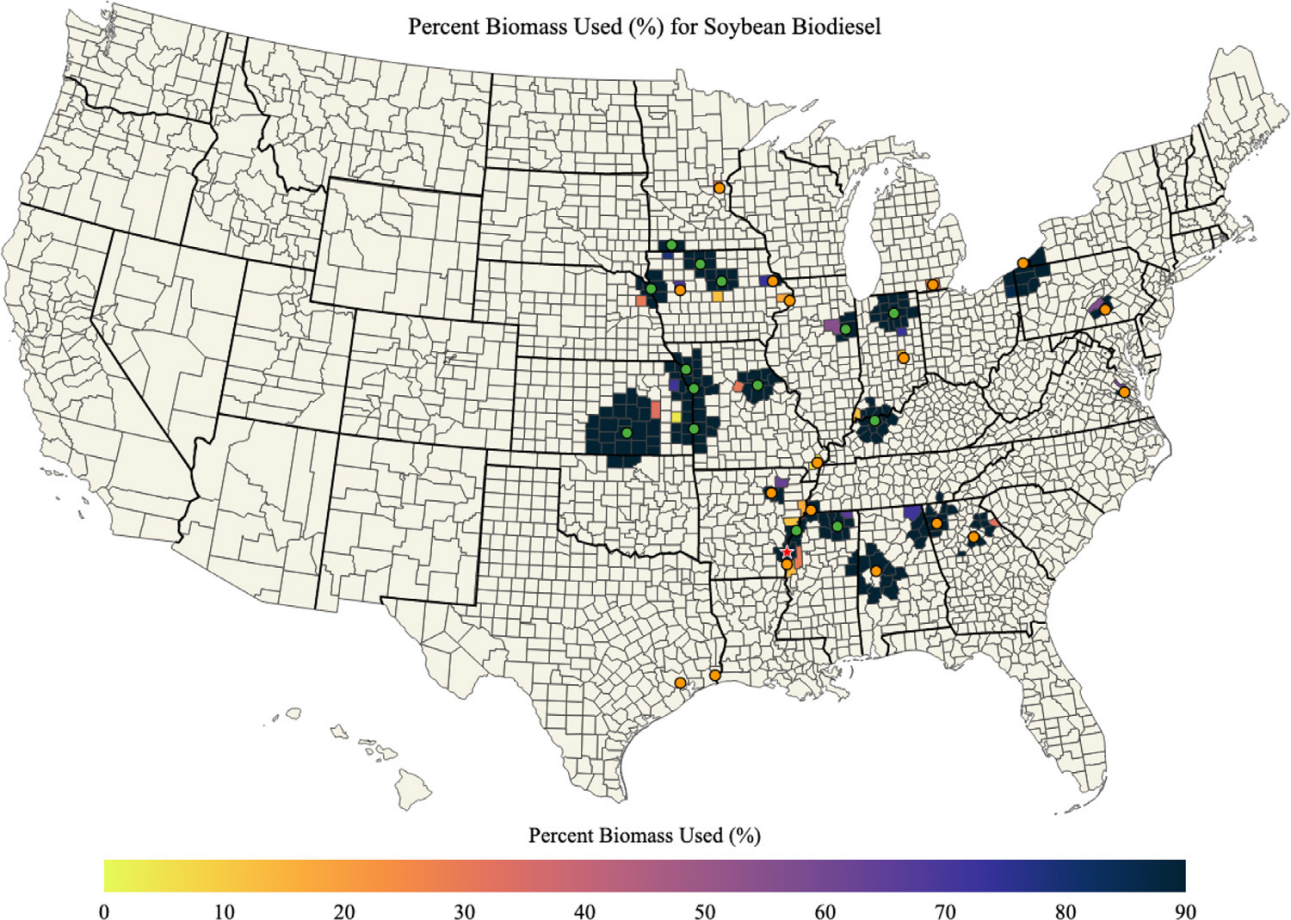
Percent of soybean biomass used for soybean biodiesel in each county of the US.

**Fig. 10:**
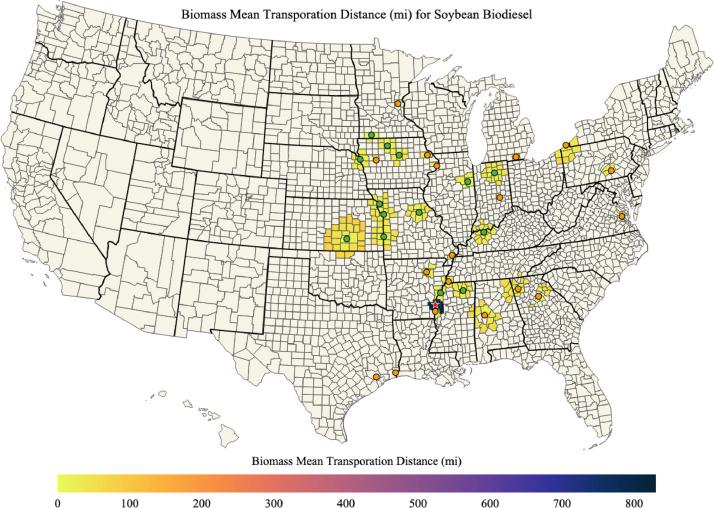
Mean transportation distance (mi) for soybeans used for soybean biodiesel in each county.

Maps of soybean biomass available, soybean biomass used for soybean biodiesel, percent of soybean biomass used for soybean biodiesel, and the mean transportation distance for soybean biomass used for soybean biodiesel in each county of the US are shown in Fig. 7, Fig. 8, Fig. 9, Fig. 10; respectively. The red star on each of the maps designates the port location chosen for barge transport of soybean biomass to the Texas biodiesel plants.

### 3.4 Validation

County-level biomass estimation was validated using reported state-level biomass-to-biofuel values. Reported values could only be found for 11 states for corn ethanol (Illinois, Indiana, Iowa, Kansas, Michigan, Minnesota, Nebraska, Ohio, South Dakota, Tennessee, and Wisconsin) and 3 states for soybean biodiesel (Indiana, Iowa, and Minnesota). Differences between the corn ethanol reported and estimated values were small with a mean difference of 1.4%. The largest discrepancy came from Indiana with a difference of 10.4%. The difference for soybean biodiesel reported and estimated values were larger. Iowa had a difference of 19.3% between the modeled and reported values (20.7% vs 40%). However, even if the three biodiesel plants in Iowa which currently produce biodiesel from various feedstocks (not exclusively soybeans) were converted to 100% soybean biodiesel, only 32.4% of the soybeans grown in Iowa would be required for biodiesel production in the state. Because of this surplus in soybean production, it is hypothesized that Iowa is exporting soybeans to other states to produce biodiesel to meet the 40% reported value, but no public information was available to confirm this hypothesis. Due to the lack of information about soybean use for biofuels, it was assumed that the high accuracy of the corn ethanol results was likely to be an accurate method for soybeans as well.

## 4 Limitations

As mentioned previously, limited information is available regarding biomass sourcing for biofuels in the US. Therefore, the assumptions made to generate this dataset could lead to inaccurate data in data limited geographic regions. However, this dataset was developed with the intention of providing a first-of-its-kind estimate of biomass sourcing for biofuel production and create a foundation for others to build upon. It is recommended that case studies be performed on the county and state level biomass sourcing to increase the fidelity of this dataset and crop producers are encouraged to increase transparency of the destination of their crops. This would allow researchers and stake holders to evaluate supply chain risks and calculate biofuel costs and environmental impact more accurately. Additionally, alternative modelling and analyses to those performed herein can be addressed with the public availability of the dataset.

## Ethics statement

The authors have read and follow the ethical requirements for publication in Data in Brief and confirming that the current work does not involve human subjects, animal experiments, or any data collected from social media platforms.

## CRediT authorship contribution statement

**Braden J. Limb:** Conceptualization, Methodology, Software, Validation, Formal analysis, Investigation, Resources, Data curation, Writing – original draft, Visualization. **Jack P. Smith:** Conceptualization, Methodology, Investigation, Resources, Writing – review & editing. **Steven J. Simske:** Conceptualization, Resources, Supervision, Writing – review & editing, Funding acquisition. **Jason C. Quinn:** Conceptualization, Methodology, Project administration, Resources, Supervision, Writing – review & editing, Funding acquisition.

## Data Availability

Geospatial Dataset Estimating Corn and Soy Inputs for Biofuel Production (Original data) (Zenodo).
